# Correction to “The abstraction of potentially zoonotic SARS‐like coronavirus (BtSY2): A threat to global health”

**DOI:** 10.1002/hsr2.1753

**Published:** 2023-12-14

Agrawal V, Khulbe Y, Jaiswal V, Paudel K. The abstraction of potentially zoonotic SARS‐like coronavirus (BtSY2): a threat to global health. *Health Sci Rep*. 2023;6:e1590. doi:10.1002/hsr2.1590

The affiliation of the corresponding author, Kusum Paudel, was mistakenly listed as “Institute of Medicine, Tribhuvan University Teaching Hospital, Kathmandu, Nepal.” The correct affiliation is “Kathmandu University School of Medical Sciences, Dhulikhel, Nepal.”

We apologize for this error.

